# Factors associated with the desire for orthodontic treatment among Brazilian adolescents and their parents

**DOI:** 10.1186/1472-6831-9-34

**Published:** 2009-12-18

**Authors:** Leandro S Marques, Isabela A Pordeus, Maria L Ramos-Jorge, Cid A Filogônio, Cintia B Filogônio, Luciano J Pereira, Saul M Paiva

**Affiliations:** 1 Department of Pediatric Dentistry and Orthodontics, Faculty of Dentistry, Federal University of Minas Gerais, Av Antônio Carlos 6627, Belo Horizonte, MG, 31270-901, Brazil; 2 Department of Orthodontics, Faculty of Dentistry, University of the Rio Verde Valley, Av Castelo Branco 82, Três Corações, MG, 37410-000, Brazil; 3 Department of Pediatric Dentistry and Public Health, Federal University of the Jequitinhonha and Mucuri Valleys, Campus II, Rodovia MGT367 5000, Diamantina, MG, 39100-000, Brazil

## Abstract

**Background:**

In the period of adolescence physical appearance takes on significant importance in the construction of personal identity, including one's relationship with one's own body. A variety of social, cultural, psychological and personal factors influences the self-perception of dental appearance and the decision to undergo orthodontic treatment. Adolescents who seek orthodontic treatment are concerned with improving their appearance and social acceptance. The aim of the present study was to determine factors associated to the desire for orthodontic treatment among Brazilian adolescents and their parents.

**Methods:**

The sample consisted of 403 subjects aged 14 to 18 years, selected randomly from a population of 182,291 schoolchildren in the same age group. The outcome variable "desire for orthodontic treatment" was assessed through a questionnaire. Self-perception of dental aesthetics was assessed using the Oral Aesthetic Subjective Impact Scale (OASIS) and the Dental Aesthetic Index (DAI) was used for clinical assessment. Statistical analysis involved the chi-square test as well as both simple and multiple logistic regression analyses.

**Results:**

The majority (78%) of the Brazilian adolescents desired orthodontic treatment and 69% of the parents reported that their children were not in orthodontic treatment due to the high costs involved. There was significant association (p ≤ 0.05) between the desire for orthodontic treatment and most types of malocclusion. However, there was no significant association between the desire for orthodontic treatment and the variables gender and age.

**Conclusions:**

The following were considered factors associated to the desire for treatment: upper anterior crowding ≥ 2 mm and parents' perception of their child's need for treatment.

## Background

Dissatisfaction with one's dentofacial appearance, recommendations from a dentist, concern on the part of parents and the influence of schoolmates who wear braces are among the main factors directly involved in the demand for orthodontic treatment [1-3]. Gender, age, intellectual level, social class, severity of the malocclusion, dental care and self-perception of facial aesthetics have also be found to be associated to the desire for orthodontic treatment [4-6]. The influence of these factors depends on the cultural and social characteristics of each subgroup of the population [4].

Understanding the factors involved in the demand for orthodontic treatment in a particular population enables a better planning of resources as well as a better assessment of treatment needs and priorities [2,3,7]. Malocclusion can be considered a public health problem due to its high prevalence and prevention/treatment possibilities. A number of studies have been demonstrated its impact on quality of life [3,8,9] and it has been considered the third highest oral health priority by the World Health Organization [10].

The association between malocclusion/treatment need and quality of life remains a controversial issue. Recent studies have revealed conflicting evidence due to differences in study designs, the population studied and assessment methods of social and psychological aspects [11,12]. However, patients who seek orthodontic treatment are undoubtedly concerned with improving their appearance and social acceptance. Thus, enhancing these aspects may be important to public healthcare.

In countries such as Brazil, the majority of scientific research developed on malocclusions in children and adolescents has mainly been limited addressing diagnostic and biomechanical aspects, whereas malocclusion as a public health problem remains poorly studied.

Thus, the aim of the present study was to determine factors associated to the desire for orthodontic treatment among Brazilian adolescents and their parents.

## Methods

A cross-sectional study was carried out in public and private schools in the city of Belo Horizonte (Brazil) between March and July 2007. The sample calculation considered an 87.7% prevalence of the desire for orthodontic treatment [3] and a maximum tolerable error of 4.5%, with a statistical power of 80%. The minimal sample size was determined to be 204 adolescents. In order to compensate for possible losses during the data collection, the decision was made to raise the sample size by 20%, totaling 245 adolescents. Additionally, a correction was performed by multiplying the sample size by 2 (Double-Stage Sampling), with the final sample size totaling 490 adolescents.

Participants were selected randomly from a population of 182,291 students in the same age group (14 to 18 years) and no prior history of orthodontic treatment. Distribution was determined proportionally to the actual distribution of schoolchildren throughout the city. First, a simple random selection by lots of one public and one private school was performed for each of the nine health districts, totaling 18 schools. Then, a random selection by lots of the participants was also performed.

A secondary random sample composed of 490 adolescents who wore or had worn orthodontic appliances was also interviewed. The objective of the interview was to determine the average cost of orthodontic treatment and whether treatment was carried out by a professional specialist or general clinical dentist. The selection criterion for this sample was by lots, carried out in the classroom on the same occasion as the selection by lots of the adolescents in the main sample.

The data collection was based on normative and subjective criteria related to biological (age, gender, malocclusion type), psychological (oral perceptions) and socioeconomic variables.

The desire for orthodontic treatment was the dependent variable, recorded on a structured questionnaire to which the adolescents responded. For the analysis of specific types of malocclusion and normative need for orthodontic treatment, the criteria of the Dental Aesthetic Index (DAI) were adopted [13]. For DAI, 10 occlusal traits were assessed and a score was obtained using the equation: 6×(missing visible teeth) + crowding + spacing + 3×(diastema) + largest anterior maxillary irregularity + largest anterior mandibular irregularity + 2×(anterior maxillary overjet) + 4×(anterior mandibular overjet) + 4×(vertical anterior open bite) + 3×(anteroposterior molar relation) + 13. The presence of crowding was assessed by segment (without crowding, one crowded segment, two crowded segments) and by the crowding severity in millimeters (lower anterior crowding - <2 mm, ≥ 2 mm and upper anterior crowding - <2 mm, ≥ 2 mm (see Tables 1 and 2). The largest anterior maxillary irregularity was designated as "upper anterior crowding" and largest anterior mandibular irregularity was designated as "lower anterior crowding. Each adolescent was then classified as having no need (score ≤ 25), elective treatment need (score 26 to 30) or highly desirable treatment need (score ≥ 31).

**Table 1:** Bivariate analysis of the associations between desire for orthodontic treatment and types of malocclusion using the chi-square test

	Desire for orthodontic treatment		
	No n (%)	Yes n (%)	P value
**Malocclusion**			
Absent	71 (34.6)	134 (65.4)	<0.001^C^
Present	15 (7.6)	182 (92.4)	
**Missing tooth**			
None	87 (22.4)	302 (77.6)	0.047^F^
One or more	0 (0.0)	14 (100.0)	
**Anterior open bite**			
< 2 mm	84 (22.8)	284 (77.2)	0.050^C^
> 2 mm	3 (8.6)	32 (91.4)	
**Crowding**			
Without crowding	51 (36.7)	88 (63.3)	
One crowded segment	28 (19.9)	113 (80.1)	<0.001^C^
Two crowded segments	8 (6.5)	115 (93.5)	
**Upper anterior crowding**			
< 2 mm	70 (32.3)	147 (67.7)	<0.001^C^
> 2 mm	17 (9.1)	169 (90.9)	
**Lower anterior crowding**			
< 2 mm	46 (29.7)	109 (70.3)	0.002^C^
> 2 mm	41 (16.5)	207 (83.5)	
**Anterior segment spacing**			
None	70 (22.4)	243 (77.6)	0.480^C^
One or two segments	17 (18.9)	73 (81.1)	
**Median diastema**			
< 2 mm	79 (21.9)	281 (78.1)	0.615^C^
> 2 mm	8 (18.6)	35 (81.4)	
**Anterior maxillary overjet**			
< 4 mm	81 (25.4)	238 (74.6)	<0.001^C^
> 4 mm	6 (7.1)	78 (92.9)	
**Anterior mandibular overjet**			
No	85 (22.3)	297 (77.7)	0.273^F^
Yes	2 (9.5)	19 (90.5)	
**Molar relation**			
Normal	68 (27.0)	184 (73.0)	0.002 ^C^
Half cuspid	17 (12.2)	122 (87.8)	
One cuspid	1 (9.1)	10 (90.9)	

^C ^Chi-square test

^F ^Fisher exact test

**Table 2:** Univariate and multivariate logistic regression analyses considering associations between the desire for orthodontic treatment (dependent variable) and remaining variables (independent)

	Non-adjusted OR (95% CI)	p	Adjusted OR * (95% CI)	p
**Malocclusion**				
Absent	1			
Present	6.4 (3.5-11.7)	<0.001		
**Open bite**				
< 2 mm	1			
> 2 mm	3.1 (0.9-10.6)	0.062		
**Crowding**				
without crowding,	1			
one crowded segment,	2.3 (1.4-4.0)	0.002		
two crowded segments	8.3 (3.8-18.4)	<0.001		
**Lower crowding**				
<2 mm	1			
≥ 2 mm	2.1 (1.3-3.4)	0.002		
**Upper crowding**				
<2 mm	1		1	
≥ 2 mm	4.7 (2.7-8.4)	<0.001	2.3 (1.1-4.6)	0.021
**Upper overjet**				
<4 mm	1			
≥ 4 mm	4.4 (1.8-10.5)	0.001		
**Molar relation**				
Normal	1			
Half cuspid	2.6 (1.5-4.7)	0.001		
One cuspid	3.7 (0.5-29.4)	0.217		
**Normative need (DAI)**				
No need	1			
Elective	3.9 (2.0-7.7)	<0.001		
Highly desirable	16.4 (5.0- 53.7)	<0.001		
**Need for treatment (parents)**				
No	1		1	
Yes	29.1 (15.4-54.8)	<0.001	21.6 (10.6-44.1)	<0.001
**Aesthetic self-perception (OASIS)**	1			
Positive	10.4 (5.3-20.5)	<0.001		
Negative				
**Parents' perception (aesthetics of child)**	1			
Satisfactory	14.1 (5.0-39.4)	<0.001		
Unsatisfactory				
**Economic level**				
High	1			
Intermediate	4.1 (2.4-7.0)	<0.001		
Low	6.2 (2.8-13.8)	<0.001		
**Father's schooling**				
High	1			
Low	4.0 (2.1-7.6)	0.001		
**Mother's schooling**				
High	1			
Low	3.9 (2.1-7.4)	<0.001		

*Adjusted for gender, age and socioeconomic level

Clinical examinations were performed by an orthodontist (CABF) who had undergone previous training and a calibration exercise for the identification of the types of malocclusion. Intra-examiner agreement for each type of malocclusion was satisfactory (maximal and minimal Kappa values were 1.00 and 0.75, respectively). The adolescents were examined on a predetermined time schedule in a room offered by the school. Necessary instruments and materials were packaged and sterilized in a sufficient quantity for each day of work.

Self-perception of dental aesthetics was assessed using the Oral Aesthetic Subjective Impact Scale (OASIS) [14]. The final OASIS result is obtained through the sum of the answers on the questionnaire, added to the value of the photograph selected on the Aesthetic Component of the Index of Orthodontic Treatment Need (IOTN-AC) so as to obtain a single score which varied from 6 to 45. This variable was dichotomized by the median into 0 = positive self-perception (OASIS<14) and 1 = negative self-perception (OASIS>14). Demographic data were investigated using a questionnaire directed at the adolescents. Parents also responded to a questionnaire that investigated their perception of their child's dental aesthetics (0 = satisfactory, 1 = unsatisfactory) and the need for orthodontic treatment (0 = No, 1 = Yes). The socioeconomic status was determined using the Brazilian Economic Classification Criterion of the Brazilian Association of Research Companies [15,16]. This criterion includes a series of questions related to the possession of household items such as a WC, radio, television, washing machine, car, full-time housekeeper, father's schooling and mother's schooling. The scores for this variable were: 0 = high (average monthly income - $4,905); 1 = intermediate (average monthly income - $1,115); and 2 = low (average monthly income - $230) [15].

All instruments used in this study were previously used in two other studies by Marques et al. [3,7]. However, they were newly tested in a pilot study with 80 adolescents (participants in the pilot study were not included in the sample of the main study). Associations between the dependent variable and independent variables were tested using univariate analysis (chi-square test and Fisher's exact test) as well as both simple and multiple logistic regression analysis. A lack of association between the variables was considered the null hypothesis (significance value greater than 0.05). Ethical approval was obtained from Human Research Ethics Committee of the University of the Rio Verde Valley (UNINCOR). Only those children who agreed to participate and whose parents signed a consent form were enrolled in the study.

## Results

A total of 403 (82%) of the 490 adolescents and their respective parents agreed to participate in the study. The reasons for the loss of 18% were the non-authorization of the parents and absence from school on the examination days. The sample was made up of 142 (35%) boys and 261 (65%) girls; 203 (50%) were between 14 and 15 years of age and 200 (50%) were between 16 and 18 years of age. Three hundred fourteen adolescents (78%) expressed a desire to receive orthodontic treatment. Among these individuals, 226 (72%) believed that orthodontic treatment could improve their lives. Forty-one percent of the adolescents considered it easier to get a job and 27% thought it easier to find a romantic partner if they wore orthodontic appliances. The desire for orthodontic treatment was also associated with the following: status or trend (22%), discrimination when smiling on the part of schoolmates (12%) and parents' use of orthodontic appliances (3%).

Among the parents, 290 (72%) considered it necessary for their child to wear an orthodontic appliance. Among these, 200 (69%) reported that the children were not in treatment due to the high costs involved. Thirty-five percent considered the dentofacial appearance of their children to be bad or very bad.

Parents of adolescents in treatment or those having already undergone treatment (secondary sample) reported an average cost of treatment of $1650 over an approximately 36-month period. Only 20% of these parents were able to state whether the healthcare professional responsible for their child's treatment was a specialist in orthodontics.

Table 1 shows a significant association (p ≤ 0.05) between the desire for orthodontic treatment and most types of malocclusion represented by the Dental Aesthetics Index. There was no significant association between the desire for orthodontic treatment and the variables gender and age (Table 3). The results of the univariate and multivariate logistic regression analyses indicate that the factors directly involved in the desire for orthodontic treatment were upper anterior crowding (≥ 2 mm) and parents' perception of their child's need for treatment (Table 2).

**Table 3:** Bivariate analysis of the associations between the desire for orthodontic treatment and biopsychosocial variables

	Desire for orthodontic treatment		
	No n (%)	Yes n (%)	P value*
**Normative need (DAI)**			
No need	71 (34.6)	134 (65.4)	
Elective	12 (11.9)	89 (88.1)	<0.001
Highly desirable	3 (3.1)	93 (96.9)	
**Need for treatment (parents)**			
No	67 (64.4)	37 (35.6)	<0.001
Yes	17 (5.9)	273 (94.1)	
**Aesthetic self-perception (OASIS)**			
Positive	75 (37.7)	124 (62.3)	
Negative	11 (5.5)	190 (94.5)	
**Parents' perception (aesthetic of child)**			
Satisfactory	80 (30.5)	182 (69.5)	<0.001
Unsatisfactory	4 (3.0)	128 (97.0)	
**Gender**			
Male	33 (23.2)	109 (76.8)	0.552
Female	54 (20.7)	207 (79.3)	
**Age**			
14 - 15 years	40 (19.7)	163 (80.3)	0.354
16 - 18 years	47 (23.5)	153 (76.5)	
**Economic level**			
High	54 (39.4)	83 (60.6)	
Intermediate	25 (13.7)	157 (86.3)	<0.001
Low	8 (9.5)	76 (90.5)	
**Father's schooling**			
≥ 8 years	68 (28.9)	167 (71.1)	<0.001
< 8 years	13 (9.2)	129 (90.8)	
**Mother's schooling**			
≥ 8 years	69 (28.5)	173 (71.5)	<0.001
< 8 years	13 (9.2)	128 (90.8)	

* Chi-square test

## Discussion

The results of the present study reveal that the majority of the Brazilian teenagers (78%) would like to receive orthodontic treatment. Seventy-two percent believed that treatment could improve their quality of life. However, 69% were unable to enjoy the benefits of treatment due to the financial costs involved. The average cost of orthodontic treatment in the city of Belo Horizonte over an approximately 36-month period is $1,650, which is lower than the value established by the Regional Dentistry Council (approximately $2,415). This represents a monthly expenditure of $46, which is equivalent to approximately 20% of the monthly income of a family with low buying power. In this context, one factor to be highlighted is that a large part of orthodontic treatment offered to the Brazilian population is being performed by clinical dentists having undergone no specialization course. Furthermore, there are no studies providing evidence on the quality of treatment performed by these dentists. There is a desire and concern on the part of the population to gain access to orthodontic treatment. However, there are only 320 specialists in orthodontics in the city of Belo Horizonte and public healthcare services offered to the population do not offer such treatment.

The results of the present study also reveal that dentofacial aesthetics is the main determinant factor in the demand for orthodontic treatment. Upper anterior crowding ≥ 2 mm was the only occlusal characteristic that influenced the desire for orthodontic treatment. This finding is supported by a number of studies carried out on other populations [3,6,11] suggesting that the negative psychosocial effects of upper anterior crowding undergo less of an influence from cultural and social standards. Although described as factors associated to the demand for orthodontic treatment in other studies [17-19], conditions such as lower anterior crowding, median diastema and maxillary overjet ≥ 4 mm were not found to be determinants in the present study. This suggests that Brazilian adolescents are more tolerant to the aesthetic effects of these conditions than European adolescents. An Australian study found that functional aspects (difficulty chewing or speaking) were more important than aesthetic aspects (ex: crowding) in the determination of the importance of orthodontic treatment [20].

In the present study, the parents' perception of their child's need for treatment was the factor that most influenced their seeking orthodontic treatment. A number of studies have stressed parents' concern with their children's dentofacial aesthetics, regardless of the population studied [18,20-22]. The main factors that motivate parents to take their children for treatment are cost, perception of the effectiveness of the treatment and the viability of treatment. In the present study, as well as in the study of Tung and Kiyak (1998) [23], parents were found to be more concerned with aesthetic conditions than functional alterations stemming from malocclusions.

Variables such as gender and age were not found to have statistically significant differences when correlated to the desire for orthodontic treatment. This finding suggests that there is full identification with peers among adolescents as well as the quest for identity, sexual satisfaction and a place in society [24]. However, other studies have pointed out that female adolescents are more critical and concerned with their dentofacial appearance than male adolescents [3,19,21,25,26]. These conflicting findings may have occurred due to different methods employed, different study designs, different variables selected and different sample sizes as well as the cultural and social characteristics of the population studied.

An epidemiological analysis of the incidences of the main oral health problems in Brazil has evidenced an enormous lack of data related to malocclusion. This is due to the accumulated treatment needs of the problems of caries and periodontal disease, an issue that is strongly correlated to the current healthcare model as well as to the inequality in access to healthcare services. Thus, those responsible for planning orthodontic treatment in both the public and private sector should concern themselves with the desires of the community as well as with the large body of evidence that supports the importance of facial characteristics in the lives of individuals.

## Conclusions

The following were considered factors associate to the desire for treatment: upper anterior crowding ≥ 2 mm and parents' perception of their child's need for treatment.

## Competing interests

The authors declare that they have no competing interests.

## Authors' contributions

LSM, CBF and SMP conceptualized the rationale and design of the study. LSM, MLRJ, CBF and LJP contributed to the collection of data, statistical analysis and interpretation of the data. LSM, IAP, MLRJ and SMP drafted the manuscript. All authors read and approved the final manuscript.

## Pre-publication history

The pre-publication history for this paper can be accessed here:

http://www.biomedcentral.com/1472-6831/9/34/prepub
